# Scenario Method of Strategic Planning and Forecasting the Development of the Rural Economy in Agricultural Complex

**DOI:** 10.1155/2020/9124641

**Published:** 2020-05-26

**Authors:** Milyausha T. Lukyanova, Vitaliy A. Kovshov, Zagir A. Galin, Zariya A. Zalilova, Eugene V. Stovba

**Affiliations:** ^1^Federal State Budgetary Educational Establishment of Higher Education, Bashkir State Agrarian University, Ufa, Russia; ^2^Birsk Branch of Federal State Budgetary Educational Establishment of Higher Education, Bashkir State University, Birsk, Russia

## Abstract

The purpose of the study is to justify the use of the universal scenario method of strategic planning and forecasting the development of the agroindustrial complex of the regional rural economy. The scientific novelty of the study lies in the application of a set of theoretical and methodological provisions for scenario planning and forecasting the development of agriculture in the regions, taking into account the assessment of their existing potential and constructing a territorial planning scheme for the priority of participation in the implementation of strategic directions of rural development in agricultural production. The paper presents a territorial model of the priority of participation of the municipal regions of the Republic of Bashkortostan in the implementation of strategic areas in the areas of development of production of grain crops, sugar beet, and oilseeds. The developed scenario method, reflecting the qualitatively heterogeneous directions of the development of enterprises, is formed taking into account the achievement of the strategic goal and potential opportunities of rural areas. This allows determining strategic decisions for the further development of rural areas and integrating them into a uniform industry development strategy. The developed approach is recommended to be used as a guideline in the development of long-term programs for the development of the crop production industry, as well as for adjusting the activities of ongoing programs.

## 1. Introduction

Today, strategic planning can become a driver, the basis for ensuring the competitiveness of the development of various territorial systems, including rural areas. Unlike classical methods, the result of strategic planning is a map of the future, which visualizes the economic space and allows considering alternative methods and ways to achieve the desired result [1]. The process of municipal strategic planning has a dualistic nature, determined by the need to coordinate interest groups of key stakeholders in the mandatory interconnection of economic and social guidelines for rural development laid down in the long-term planning format [2].

The preparation of planning programs is carried out mainly for cities; at the same time, strategic planning and forecasting the development of rural territorial municipal units is no less relevant, where development plans are the organizing document aimed at ensuring their competitiveness. The lack of a comprehensive scientific justification for the formation of real methods of strategic planning and forecasting the development of rural areas can lead to the adoption of incorrect management decisions, which will make it difficult to determine priorities in strategic development programs of rural municipalities [3].

The problems of the relationship between cities and rural territories of European regions, and in particular, on the example of the Calabria region (southern Italy), are considered by foreign scientists. The districts are faced with economic, social, and environmental problems, which lead to unemployment, depopulation, and fragmentation of available capital. Institutional and decision-making bodies, in particular at the local level, are confronted with the difficulty of choice, which requires the availability of potential for decision-making processes and tools that can integrate the use of territorial resources [4].

In an uncertain socioeconomic and climatic context, the achievement of sustainable agricultural production is a serious problem for both producers and agricultural consultants. Therefore, various options are proposed for the development of decision support tools that will be useful for developing and evaluating new production strategies in accordance with the sustainability of farms and environmental protection [5].

For many African countries, the structure of the economy is focused on agriculture and agribusiness. Recently, investments in agriculture have been considered as one of the key ways to strengthen the economies of African countries and create jobs for growing youth. However, the form of agriculture that will achieve these goals cannot be based on typical agricultural practices in many parts of Africa. Various scenarios based on science, research, and innovations adapted to African conditions take into account the combination of dominant factors developed by African experts [6].

In the scientific literature, adaptive strategies for the development of the agroindustrial complex are developed, implemented in various short-term and long-term prospects. The possible adaptation strategies are developed on the basis of the analysis of the general picture of global trends and challenges, opportunities, and threats that interact with country-specific structural factors [7]. Recommendations on the national scientific and technical policy for three main sectors of the agroindustrial complex are proposed: crop production, animal husbandry, and the food industry. They also highlight measures to reduce institutional barriers to increase the investment attractiveness of the industry [8]. Among the key strategic tasks are intensifying agricultural production, overcoming the shortage of human resources, adapting the development of agricultural infrastructure to modern realities, and maintaining the multilayered nature of the industry [9]. The main trends of the transformation of the functional-territorial structure of agriculture and the influence of processes of agroindustrial integration are analyzed [10]. Methodologies are proposed on the example of agriculture and the food sector, which take into account the coincidence over a certain period of time between scientific and strategic documents, and thus the relationship between science and strategy was explored [11]. The structure of the strategy is proposed based on three objectives: to predict the future of the food system, to agree on the most important drivers of change affecting food security, and to reach a consensus on the forecast for the period until 2030 [12].

The analysis of the differences between the factors (endogenous and exogenous) that potentially affect the competitiveness of the Italian agriculture, and their influence on the economic performance of agriculture at the provincial level, was the purpose of the study of Italian scientists. They identified the main characteristics of Italian agricultural systems and basic components using data collected during the last Italian census of agriculture in 2010 at the provincial level. The results were used as explanatory variables in regression models to assess their relationship with agricultural productivity indicators and provincial performance indicators [13].

The spatiotemporal changes and dynamic characteristics of modern conditions for the development of agriculture in China were studied. A system of indicators was put forward for the coherence of agricultural natural factors and regional functions, the principles of regionalization, and the scientific method of modern agrarian regionalization against the backdrop of new development. Using cluster and qualitative analysis, they advanced a modern scheme of regionalization of agriculture in China, which divides China into 15 agricultural regions of the first class and 53 agricultural subregions [14].

The research was also conducted in the field of collective action, taking into account the territorial aspect, which is based on a synthesis of research in the field of rational use of natural resources, agriculture, food supply, land use planning, and development of rural and suburban areas [15].

The presence of a large number of state agricultural lands allows the municipality to promote innovative types of agriculture in connection with the development of the cities of Lausanne. The studies have contributed to the modern understanding of the recognition of agricultural land as part of the public interest, as well as the implementation of agricultural and food policies at the territorial level [16].

The formation of a broad view of the countryside, in a territorial context, stimulating the diversification of the economy, was considered in the documents of the World Bank. The result of which is the assessment and modeling of material factors adopted in rural areas and actions that affect the practice of the local population and require new institutional and market control in relation to the environment [17].

Much attention is paid to the consideration of terminological issues of the territorial impact of other sectoral strategies on agriculture in the countries of the European Union [18]. The agricultural sector is the main source of income for approximately 20% of the EU population. In addition, the combined agricultural and food sector is an important part of the EU economy, accounting for 15 million jobs (8.3% of total employment) and 4.4% of GDP. Today, 12 million active farmers across Europe have an average farm size of about 15 hectares and are expected to meet the needs of 500 million Europeans [19].

The institutional mechanisms in the Amazon are considered taking into account the structural diversity characterizing the rural sector in the region. On the basis of various models of interconnections between technological trajectories, two large representative configurations of development conditions are established: (1) rural economy based on agriculture and animal husbandry and (2) an economy based on agroforestry-ameliorative systems [20].

The international discussion of structural change continues to focus on sectoral approaches. The discussion of rural transformation is most often limited to long-standing biases. To solve the main problems associated with their demographic and economic transition, many developing countries need to consider the new development dynamics of rural and urban areas and adopt territorial development strategies [21, 22].

The use of strategic development mechanisms will enable local authorities to assess the potential and reserves of rural territories, taking into account unique features, shape their future image, and develop practical measures to achieve this image. From the point of view of the image component, thanks to the development strategy and planned future, the image of rural areas will improve, which, in turn, will directly affect their investment attractiveness.

Despite the availability of numerous works on this issue, there are problems in the methodology for predicting the development of the agricultural system in the region, taking into account the interconnections of all elements and the need for a comprehensive study of the features of agricultural production with a systematic consideration of intellectual, innovative, social, and other characteristics. The comprehensive analysis of scientific publications allows us to conclude that the problem of choosing a methodology for making forecasts, especially for the long term, is still far from being solved. There are difficulties in applying methods of processing a significant set of information characterizing the development of agriculture. In this regard, the research and development of the scenario method for forecasting the development of agriculture in the regions have significant scientific theoretical significance and practical orientation.

The insufficient development of organizational mechanisms for strategic planning of the development of individual municipalities has a negative impact on the functioning of the countryside. The absence of clearly formulated strategic programs for the socioeconomic development of rural territories of many regions strengthens the polarization among municipalities in the framework of the dichotomy “city—periphery (rural area)” and widens the gap in living standards between the urban and rural population of the region. Assessment of the socioeconomic situation in rural areas of the region shows the imbalance of the social sphere and the growth of social tension [20].

The purpose of the study is to justify the use of the universal scenario method of strategic planning and forecasting the development of the agroindustrial complex of the regional rural economy.

The study was conducted on the example of one of the largest agricultural regions of Russia—the Republic of Bashkortostan. The use of the scenario method in strategic planning for the development of the agroindustrial complex of the agricultural sector will help bring the rural municipalities of the region to a qualitatively new level of socioeconomic development. The results of the study will allow the formation of a single platform for strategic planning for the development of the plant-growing industry in the region and will be the scientific basis for the development of a roadmap and strategy for the development of rural territories of the Republic of Bashkortostan for the period until 2030 [11]. By analogy, the main methodological approaches and research results can be used in any region of the world with similar development conditions.

## 2. Data and Methods

**Figure 1:**
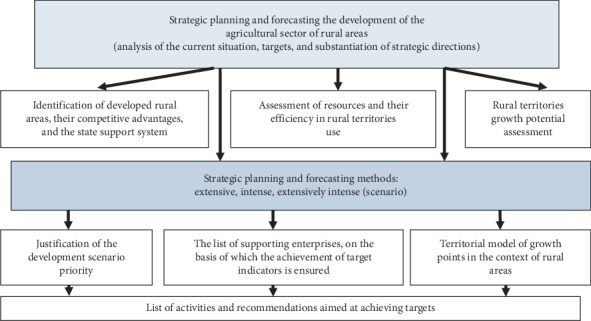
Methodology of strategic planning and forecasting the development of the agroindustrial complex of rural territories (Source: developed by the authors).

To achieve this goal, a methodology for strategic planning and forecasting the development of the agroindustrial complex of rural territories was developed (Figure 1).

The results of the study were determined using the scenario method, which is based on the development of agricultural development scenarios, covering all possible options for realizing the internal potential and the impact of external conditions. In the framework of the implementation of the scenario method, the following development scenarios are proposed—extensive, intensive, and extensive-intensive scenarios.

The extensive scenario, on the one hand, creates opportunities for the growth of rural economies, as it increases production resources, and on the other hand, it limits the possibilities of qualitative improvement of the economy. This scenario does not provide for a significant change in the quality parameters of agricultural production and provides for certain measures of state support and regulation of agricultural production.

The intensive scenario, which is the main form of expanded reproduction, is carried out by improving the system of conducting the industry on the basis of scientific and technological progress to increase the output per area unit, increase labor productivity, and reduce costs per unit of the output. An intensive scenario for the development of the agroindustrial complex implies substantial state support.

The extensive-intensive scenario provides comprehensive development of the regional economy of rural areas. Production volumes and other targets were determined on the basis of a combination of quantitative indicators for the extensive scenario and qualitative indicators for the intensive development scenario. The implementation of this scenario is impossible without significant direct state support.

The results of the study were determined using the scenario method (extensive-intensive scenario), which is based on the development of scenarios for the development of the crop sector, covering all possible options for realizing the internal potential of rural areas and the impact of external conditions.

Based on these indicators, we calculate the forecast values of crop production for 2019–2030 according to the following formula:

(1) $$\begin{array}{c} В\prod =a+b*S_{k}, \end{array}$$

where *B*∏ is the forecast values of crop production, a and *b* are the coefficients characterizing the relationship between the studied variables, and *S*_*k*_ is the forecast values of sown areas of crops in the *k*th forecast period.

The coefficients a and *b* are calculated by the following formulas:

(2) $$\begin{array}{c} b=\frac{\sum_{i=1}^{n}S_{i}*B\prod_{i}-n*\bar{S*B\prod }}{\sum_{i=1}^{n}S_{i}^{2}-n*\bar{S^{2}}},\\ a=\bar{B\prod }-b*\bar{S}, \end{array}$$

where *n* is the number of measured values, $\bar{B\prod }$ is the average value of crop production, and $\bar{S}$ is the average sown area of crops.

To calculate the predicted value according to formula (1), we calculate the coefficients *a* and *b*:

 
*b* grain crops = (59572369.6 + 67548209.6 + 53531275.0)) − 180538377.9)/(9463205.3 − 9462172.8) = (180651854.2 − 180538377.9)/1032.5 = 113476, 3/1032.5 = 109.9
 
*b* sugar beets = (410193.8 + 702751.1 + 360714.6)) − 1447872.1)/(3973.1 − 3866.4) = (1473659.5 − 1447872.1)/106.7 = 25787, 4/106.7 = 241.8
 
*b* oil crops = (52044.3 + 707014.0 + 791459.2)) − 2012106.5)/(150030.1 − 149767.4) = (2018916.5 − 2012106.5)/262.7 = 6810, 0/262.7 = 25.9
 
*a* crops = 33885.5 − 109.9 ∗ 1776.0 = −161296.1
 
*a* sugar beets = 13443.6 − 241.8 ∗ 35.9 = 4762.3
 
*a* oil crops = 3001.8 − 25.9 ∗ 223.4 = −2790.5

The calculation of the correlation coefficient determines a close relationship between the considered indicators and the accuracy of the forecast:

(3) $$\begin{array}{c} r=\frac{\sum_{i=1}^{n}S_{i}*B\prod_{i}-n*\bar{S*B\prod }}{\sqrt{\left(\sum_{i=1}^{n}S_{i}^{2}-n*\bar{S^{2}}\right)*\left(\sum_{i=1}^{n}В\prod_{i}^{2}-n*\bar{В\prod^{2}}\right)}}. \end{array}$$

Here is the calculation of the correlation coefficient and its level:

(4) $$\begin{array}{c} r_{\text{grain crops}}=\frac{\left(180651854.2-180538377.9\right)}{\sqrt{\left(9463205.3-9462172.8\right)*\left(3471494203.8-3444674553.7\right)}}=\frac{113476.3}{166409.1}=0.68,\\ r_{\text{sugar beets}}=\frac{\left(1473659.5-1447872.1\right)}{\sqrt{\left(3973.1-3866.4\right)*\left(555592390.2-542188454.2\right)}}=\frac{25787.4}{37807.4}=0.68,\\ r_{\text{oil crops}}=\frac{\left(2018916.5-2012106.5\right)}{\sqrt{\left(150030,1-149767,4\right)*\left(27610327,1-27032409,7\right)}}=\frac{6810,0}{12321,2}=0,55. \end{array}$$

The correlation coefficient (0.68) indicates a strong relationship between the considered indicators.

Based on the developed methodology, the assessment was made of the strategic potential of the main crops cultivated in the Republic of Bashkortostan. Based on the information on the republican indicators of the agroindustrial complex by calculating their relationship, the predicted data of the strategic development of the economy of rural areas of the region were obtained.

## 3. Results

In modern conditions of socioeconomic transformations in rural areas, the development of new scientific approaches is required in relation to what constitutes the reproductive basis for the development of rural territories. When developing a development strategy for the agroindustrial complex, it is necessary to apply measures to substantiate the option for the effective functioning of rural municipal districts for the long term.

Let us make a forecast of the gross production of the main crops for 2019–2030. The prediction based on these indicators is based on the assumptions about the correspondence of the cultivated area increase in gross harvest and the invariance of seasonal dynamics. At the same time, the actual gross yield of the previous year is adjusted annually for the indicators under consideration, the main of which is the crop production index. Sown areas, productivity, and gross yield in the Republic of Bashkortostan for 2016–2018 are given in Table 1.

From Table 1, we can conclude that for 2016–2018 there is a decrease in the gross harvest of grain crops by 8% due to a decrease in the sown area by 2.4% and productivity by 5.8%. Despite the increase in cultivated areas for growing of sugar beets by 33.4 thousand hectares, due to a decrease in yield, a gross harvest reduction of 21% is observed. A significant increase in the gross yield of oilseeds (sunflower) was affected by both the expansion of sown areas (5.8%) and the increase in yield (35.8%).

To predict the indicator of sown areas of agricultural crops until 2030, we use the “Prediction” function in Excel and the obtained values are shown in Table 2.

The forecast values of grain production for 2019–2030, calculated according to formula (1), have the following form:

 
*В*∏_2019_ = −161296.1 + 109.9 ∗ 1757.8 = 31888.9 thousand centners
 
*В*∏_2020_ = −161296.1 + 109.9 ∗ 1765.6 = 32746.2 thousand centners
 
*В*∏_2025_ = −161296.1 + 109.9 ∗ 1804.6 = 37032.3 thousand centners
 
*В*∏_2030_ = −161296.1 + 109.9 ∗ 1843.6 = 41318.5 thousand centners

According to the extensive-intensive development scenario, a consistent increase in the gross harvest of grain crops in the Republic of Bashkortostan to 41.3 million centners occurs for the forecast period.

**Figure 2:**
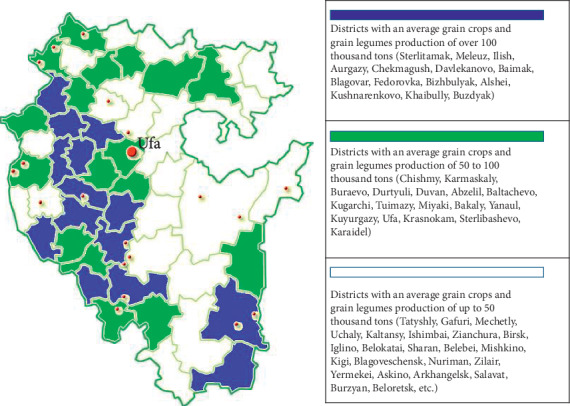
Territorial model of priority of participation in implementation strategic directions of the municipal regions of the republic in direction of development of grain production.

Based on the obtained data, we will present a zoned model, which suggests the possibility of developing the crop production industry of the Republic of Bashkortostan according to three scenarios—extensive, intensive, and extensive-intensive scenarios (Figure 2).

Agriculture of the Republic of Bashkortostan is a key point in the growth of the interregional and international competitiveness of the republic. The republic is the northern region for the production of sugar beet root crops. Sugar beet production is concentrated in the Ural steppe and the republic's southern forest-steppe [23].

The forecast values of sugar beet production for 2019–2030, calculated according to formula (1), have the following form:

 
*В*∏_2019_ = 4762.3 + 241.8 ∗ 34.8 = 13177.6 thousand centners
 
*В*∏_2020_ = 4762.3 + 241.8 ∗ 36.1 = 13491.9 thousand centners
  В∏_2025_ = 4762.3 + 241.8 ∗ 42.6 = 15063.7 thousand centners
 
*В*∏_2030_ = 4762.3 + 241.8 ∗ 49.1 = 16635.6 thousand centners

Thus, according to the extensive-intensive development scenario for the forecast period, there is a consistent increase in the gross yield of sugar beets in the Republic of Bashkortostan to 16.6 million centners.

The sustainable development of sugar beet production can be achieved through the introduction of new advanced resource-saving technologies. The main directions of increasing sustainability are the level of innovation and investment, the quality of products, qualitative changes in the management and maintenance of production, the level of competitiveness of the industry, etc. [24].

Sown areas of sugar beets in the republic are declining. If in 2000 sugar beets were cultivated on an area of 71.0 thousand ha, then in 2016, they would be cultivated on an area of 33.5 thousand ha. This is due to the relatively high production cost of root crops and, accordingly, the low competitiveness of imported raw materials and sugar (from Ukraine, Cuba, etc.) [4].

**Figure 3:**
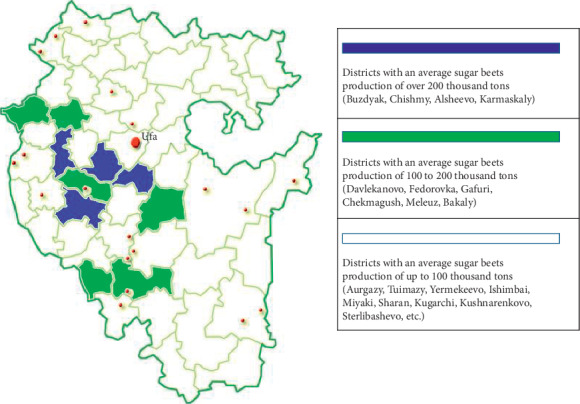
Territorial model of priority of participation in implementation strategic directions of the municipal regions of the republic in the direction of development of sugar beet production.

The territorial model of priority participation in the implementation of strategic directions of the municipal regions of the Republic of Bashkortostan in the direction of development of sugar beet production is presented in Figure 3.

Concerning world trade in oil crops, vegetable oil in recent years has increased dramatically and exceeds the volume of world trade in wheat and other crops. The presence of domestic demand from oilseed processors and the constantly growing demands on the world market make it possible to predict the stability of demand for oilseeds of domestic agricultural producers.

The forecast values of oilseed production for 2019–2030, calculated according to formula (1), have the following form:

 
*В*∏_2019_ = −2790.5 + 25.9 ∗ 230.1 = 3174.6 thousand centners
 
*В*∏_2020_ = −2790.5 + 25.9 ∗ 236.2 = 3332.8 thousand centners
 
*В*∏_2025_ = −2790.5 + 25.9 ∗ 266.7 = 4123.5 thousand centners
 
*В*∏_2030_ = −2790.5 + 25.9 ∗ 297.2 = 4914.1 thousand centners

According to the extensive-intensive development scenario, a consistent increase in the gross yield of oilseeds in the Republic of Bashkortostan to 4.9 million centners occurs for the forecast period.

**Figure 4:**
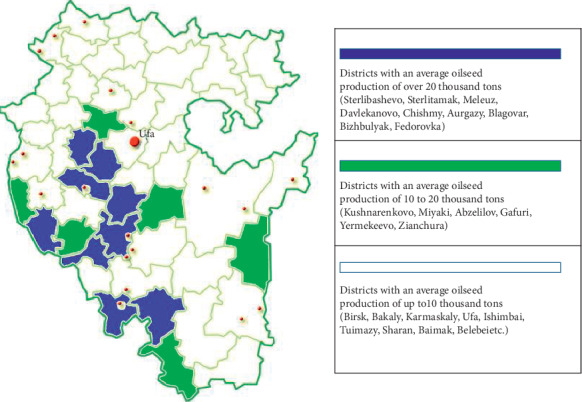
Territorial model of priority of participation in implementation strategic directions of the municipal regions of the republic in direction of development of oilseed production.

The main volumes of sunflower production are concentrated in Sterlitamak, Meleuz, Davlekanovo, Chishmy, Blagovar, and Fedorovka districts. The territorial model of priority participation in the implementation of strategic directions of the municipal regions of the Republic of Bashkortostan in the direction of development of oilseed production is presented in Figure 4.

The strategic development of the agroindustrial complex determines the priority areas of the agroindustrial complex and the tools for its regulation by the state in the medium term. This allows comprehensively approaching the development of key industries and increasing the efficiency of agricultural production in the Republic of Bashkortostan.

## 4. Discussion of the Results

The conducted scenario forecasting and analytical calculations allow us to compare the proposed methodology with models developed by other scientists and illustrate two features of agricultural development in the future.

Firstly, increasing the competitiveness of crop production can provide growth in crop production, which should be assessed as an important factor of great strategic importance. The use of this potential should become one of the main targets for the strategic development of rural areas.

Therefore, organizational and economic, technological, agricultural, and biological measures are recommended for enterprises producing grain crops, in order to increase their basic indicators for strategic development, for all the laboriousness of production [25].

To implement the strategic direction for the development of sugar beet production, it is necessary to introduce new highly productive hybrids and varieties of sugar beet that are resistant to diseases and pests, suitable for cultivation by intensive technology and cultivation of sugar beets on irrigated lands, paying special attention to increasing sugar content and technological qualities of root crops, etc. [26].

Achieving goals and fulfilling the tasks of intensifying the production of oilseeds for 2019–2030 will be ensured by expanding the raw material base through the development and implementation of modern technologies, selecting and cultivating oilseeds, optimizing the territorial distribution of the raw material base of the industry, forecasting the structure of production and consumption by developing a balance of demand and supply of vegetable oil, as well as the need for meal and meal, expanding cultivated areas of export-oriented oilseeds (rapeseed and false flax), etc. [27, 28].

Secondly, this potential can also be interpreted as an insurmountable limitation in the development of agriculture with its focus on traditional sales markets—as the natural limits of increasing the capacity of the domestic market for crop production; when approaching it, the growth rate of its production will decrease [29, 30].

Therefore, it is necessary to prepare in advance a system of state regulation tools that are adequate to the features of the future stage of agricultural development, which will allow domestic producers to enter new sales markets, as well as effectively address the socioeconomic problems of sustainable rural development in the face of an inevitable slowdown in agricultural growth production, which should also be the most important areas of the strategy for the development of agriculture.

The proposed territorial model of the agroindustrial complex priority of municipalities predetermines the format and method of setting goals for regional development strategies of rural territories. Territorial modeling, which takes into account socioeconomic and resource potential, allows you to develop development scenarios and choose the one that allows you to achieve your goals. In accordance with the developed methodology, it is advisable to develop a scientifically based integrated territorial model for placing priority areas for the development of the agricultural sector, taking into account the concept of rural development.

When implementing the model, the scenario-based approach to determining the trajectories for achieving the targets turned out to be effective (in this case, the development of African and French scientists on adapting the scenarios to the dominant external and internal conditions of the municipality is relevant) [5, 6]. The developed territorial model of priority participation in the implementation of strategic directions of the region's municipal areas based on the scenario method involves a comprehensive and flexible combination of scientific developments of leading world scientists and the best practices of strategic planning for rural development.

## 5. Conclusions

The formed scientific positions can be used in the formation of strategies, highlighting competitive advantages, trends, and strategic priorities for rural development. The developed conceptual approaches will improve the organizational and economic mechanism for developing strategic plans and programs, developing and formulating a mission, general goals, and subgoals for rural development.

The developed recommendations will improve the quality of planning and forecasting activities of municipal authorities and expand the time horizon of strategic planning in determining the parameters of the development of the crop sector in the republic. The results of the study are of practical importance in compiling and updating strategic development programs for rural areas of regions with similar socioeconomic development conditions.

To determine the strategic potential for the effective development of the crop sector, the analysis was made of all categories of farms in the Republic of Bashkortostan. The assessment of the strategic potential of the main agricultural crops cultivated in the republic, according to the extensive-intensive production development scenario, amounted to the following: grain crops, 41.3 million tons; sugar beets, 16.6 million tons; and oil crops, 4.9 million tons.

The presented territorial model of the priority of participation in the implementation of strategic directions for the development of production of grain crops, sugar beets, and oil crops makes it possible to develop the crop production industry according to extensive, intensive, and extensive-intensive scenarios.

## Figures and Tables

**Table 1:** Indicators of crop production in farms of all categories in the Republic of Bashkortostan for 2016–2018.

Indicators	Years		
	2016	2017	2018
Sown areas, thousand ha			
(i) Grain crops	1792.2	1785.7	1750.0
(ii) Sugar beets	30.1	44.1	33.5
(iii) Oil crops	211.7	234.6	224.0

Yield, centner/ha			
(i) Grain crops	18.5	21.2	17.5
(ii) Sugar beets	452.7	361.3	321.4
(iii) Oil crops	11.6	12.8	15.8

Gross production, thousand centners			
(i) Grain crops	33239.8	37827.3	30589.3
(ii) Sugar beets	13627.7	15935.4	10767.6
(iii) Oil crops	2458.4	3013.7	3533.3

Source: key indicators of agriculture of the Republic of Bashkortostan in 2018: statistical bulletin—Ufa: Bashkortostanstat, 2019—46 *p*.

**Table 2:** Predicted values of sown area agricultural crops, thousand ha.

Indicator	Years											
	2019	2020	2021	2022	2023	2024	2025	2026	2027	2028	2029	2030
Grain crops	1757.8	1765.6	1773.4	1781.2	1789.0	1796.8	1804.6	1812.4	1820.2	1828.0	1835.8	1843.6
Sugar beets	34.8	36.1	37.4	38.7	40.0	41.3	42.6	43.9	45.2	46.5	47.8	49.1
Sunflower	230.1	236.2	242.3	248.4	254.5	260.6	266.7	272.8	278.9	285.0	291.1	297.2

Source: developed by the authors.

## Data Availability

Data will be available upon request to the corresponding author.
